# Catalytic Degradation of Lignin over Sulfonyl-Chloride-Modified Lignin-Based Porous Carbon-Supported Metal Phthalocyanine: Effect of Catalyst Concentrations

**DOI:** 10.3390/molecules29020347

**Published:** 2024-01-10

**Authors:** Fangli Du, Xuequan Xian, Peiduo Tang, Yanming Li

**Affiliations:** High Performance Materials Research Institute, Guangxi Academy of Sciences, Nanning 530007, China; dfl@gxas.cn (F.D.);

**Keywords:** lignin, supported metal phthalocyanine, lignin degradation, liquid products

## Abstract

A sulfonyl-chloride-modified lignin-based porous carbon-supported metal phthalocyanine catalyst was prepared and used to replace the traditional Fenton’s reagent for lignin degradation. The catalyst underwent a detailed characterization analysis in terms of functional group distributions, surface area, morphological structure, via FT-IR, XPS, BET, and SEM. The catalyst possessed a specific surface area of 638.98 m^2^/g and a pore volume of 0.291 cm^3^/g. The prepared catalyst was studied for its ability of oxidative degradation of lignin under different reaction conditions. By optimizing the reaction conditions, a maximum liquid product yield of 38.94% was obtained at 135 °C with 3.5 wt% of catalyst and 15 × 10^−2^ mol/L H_2_O_2_; at the same time, a maximum phenols selectivity of 32.58% was achieved. The compositions and properties of liquid products obtained from lignin degradation using different catalyst concentrations were studied comparatively via GC-MS, FT-IR, ^1^H-NMR, and EA. Furthermore, the structure changes of solid residues are also discussed.

## 1. Introduction

Facing the problem of energy shortage and environmental pollution caused by excessive consumption of fossil energy, it is especially important to development and employ sustainable renewable resources such as renewable biomass resources. According to the predictions of the International Energy Agency (IEA), bioenergy will account for about 10% of total primary energy use by 2035, and by 2050, bioenergy will account for around 27% of the world’s total transport energy [1]. Thus, it can be seen that there is considerable urgency and demand for biomass renewable energy that can replace traditional fossil energy. One of the most popular approaches is to utilize industrial waste lignocellulose [2].

Lignin is the most abundant three-dimensional phenolic polymer bearing three aromatic structures: syringyl (S), guaiacyl (G), and hydroxyphenyl (H) units. Each unit has been verified to stem from one of the monolignols: p-hydroxycinnamyl, coniferyl alcohol, and sinapyl alcohol [3,4]. The three basic monolignols are mainly linked by C-C or C-O types; however, other chemical bonds are also presented in lignin such as aryglycerol-β-ether dimer (β-O-4, accounting for 45–50%), biphenyl/dibenzodioxocin (5-5′, accounting for 18–25%), pino/resinol (β-5, accounting for 9–12%), diphenylethane (β-1, accounting for 7–10%), aryglycerol-α-ether dimer (α-O-4, 6–8%), phenylcoumaran (β-β′, 0–3%), and siaryl ether (4-O-5, 4–8%) [5]. Because of this peculiar phenylpropanol structure, and the recent emergence of biorefineries, lignin attracts global attention and was thought to be a low-value energy source and a viable means to manufacture chemicals and biofuels [6]. However, various strategies have been implemented to transform lignin into small chemicals via cleavage of C-C and C-O linkages [4].

Among these strategies, liquid phase catalytic degradation of lignin to produce small chemicals has been found to be an effective measure because of the mild reaction conditions and high product selectivity. So far, numerous studies have examined the liquid phase catalytic degradation of lignin [7,8], including acid-catalyzed [9,10,11,12] and base-catalyzed depolymerization [13,14]. Although acid- and base-catalyzed depolymerization of lignin have been studied extensively, there are still some drawbacks that need to be faced; for instance, it is difficult to isolate the homogeneous acid and base catalyst after the reaction is completed, thus resulting in environmental pollution. Furthermore, carbocation occurring during acid-catalyzed depolymerization or the quinone structure formed during base-catalyzed depolymerization favor the production char, and so reduce the selectivity of aromatic monomers.

**Figure 1:**
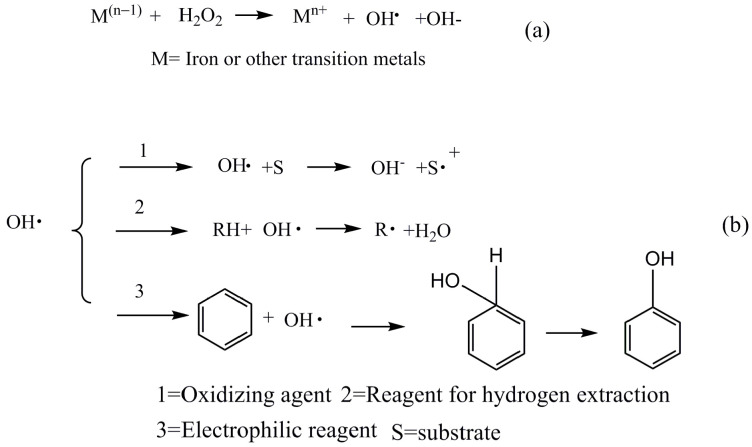
Reaction formulas: (**a**) formation of HO· from H_2_O_2_; (**b**): the reaction triggered by HO·.

In order to avoid the disadvantages of acid- or base-catalyzed depolymerization, researchers gradually started to use Fenton’s reagent for the lignin treatments. G. Bentivenga et al. found that the application of FeSO_4_·7H_2_O/H_2_O_2_ could accelerate the degradation of lignin [15]. Francesc Torrades [16] and co-workers investigated the degradation of black liquor using [Fe(II)]/[H_2_O_2_]; the best conversion of 94.8% was achieved when [H_2_O_2_] = 44.1 mM, [Fe(II)] = 4.655 mM, at 298 K and pH = 3. Phisit Seesuriyachan et al. [17] reported that only about 10% of lignin was present after the degradation using the synergistic catalysts 7.61 mg/L Fe^0^, 9.89 mg/L Fe^2+^, 14.27 mg/L Fe^3+^, and 376.88 mg/L H_2_O_2_ at pH = 3. Recently, some studies on the treatment of lignin with Fenton-like reagent have been conducted. For instance, YuYun Lin and co-workers [18] investigated the cleavage of a lignin model compound into aromatic chemicals using CuFe_2_O_4_@rGO-150 and H_2_O_2_; the best yield of guaiacol was 72.6% and the best yield of 2-methoxy-4-propyl phenol was 52.5%. In these systems, OH· was generated from the decomposition of H_2_O_2_ by Fe^2+^ (or other transition metals) catalysis at low pH (−2–3) (Figure 1a); subsequently a large number of subsequent reactions were triggered by HO· (Figure 1b) [19,20,21,22].

**Figure 2:**
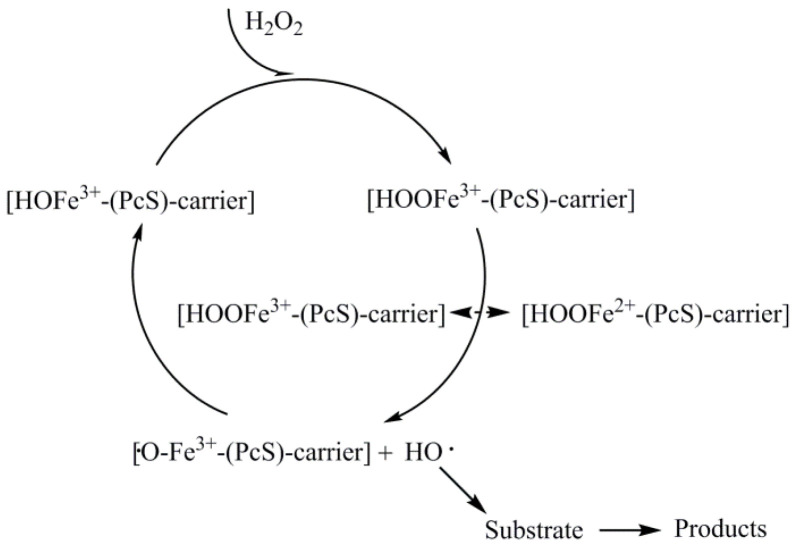
The route for HO· generation.

**Figure 3:**
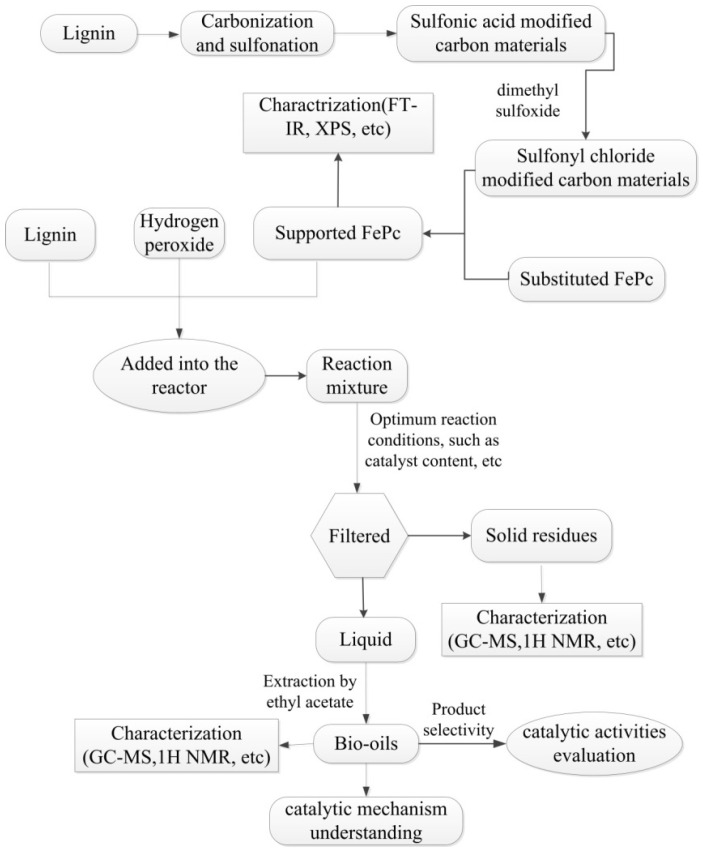
The detailed experiment process in this paper.

It can be seen from the above that Fenton’s reagent or Fenton-like reagent has feasibility in the depolymerization of lignin. In our previous literature investigation, we found that metal phthalocyanines exhibit high catalytic capacity for various types of reactions [23]. Tao X et al. found that the introduction of metal phthalocyanine can ensure the Fenton’s reagent maintains high catalytic activity even under neutral or weakly alkaline conditions [24]. In addition, in the metal phthalocyanine structure, the metal atom (M) binds to the N atom to form M-N chelate; when the metal atom M is strongly anchored, this kind of structure can avoid the metal loss and agglomeration. The above advantages of metal phthalocyanine create possibilities for its further application to replace the traditional Fenton’s reagent. In the presence of metal phthalocyanine or supported metal phthalocyanine, the generation of HO· abidesby the principle described in Figure 2 [25,26]:

Therefore, in the present study we aimed to synthesize the sulfonyl-chloride-modified lignin-based porous carbon-supported metal phthalocyanine as a Fenton catalyst to replace the traditional ferrous ion, and develop a new Fenton process for lignin degradation, taking this supported metal phthalocyanine as the catalyst and H_2_O_2_ as the oxidant. The detailed experiment process is summarized in Figure 3. This experiment injects new vitality into the development of Fenton’s reagent; additionally, the research theoretically provides evidence of the new technology for the resource utilization of lignin.

## 2. Results and Discussions

### 2.1. Characterization of Catalyst

#### 2.1.1. FTIR Analysis

**Figure 4:**
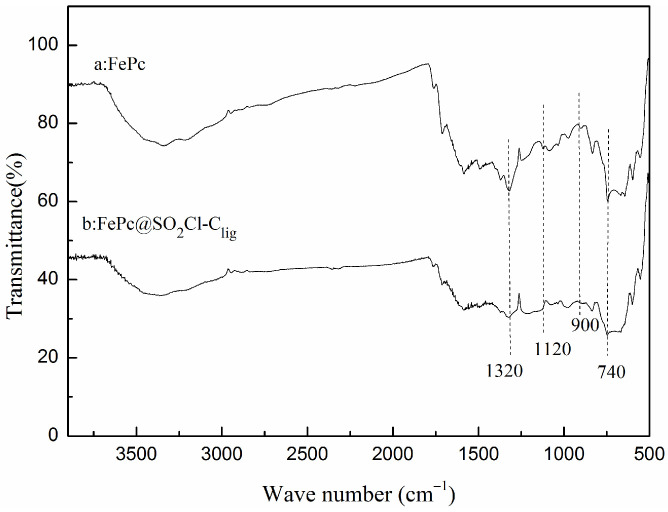
FT-IR spectra of FePc and FePc @ SO_2_Cl-C_lig_.

The structural changes of FePc and FePc@SO_2_Cl-C_lig_ were confirmed using FT-IR analyses; the results are exhibited in Figure 4.

The FT-IR spectra revealed that the characteristic peaks of FePc appeared at 1320 cm^−1^, 1120 cm^−1^, and 740 cm^−1^, which are assigned to the vibration of the phthalocyanine ring [27,28]. Similar characteristic bands were exhibited obviously in the spectrum of FePc@SO_2_Cl-C_lig_, indicating that FePc clung to the surface of SO_2_Cl-C_lig_ without changes in the chemical feature. A new weak signal derived from S-N stretching vibration appeared around 900 cm^−1^ [29], demonstrating that the FePc successfully introduced into the surface of SO_2_Cl-C_lig_ via chemical reaction.

#### 2.1.2. XPS Analysis

**Figure 5:**
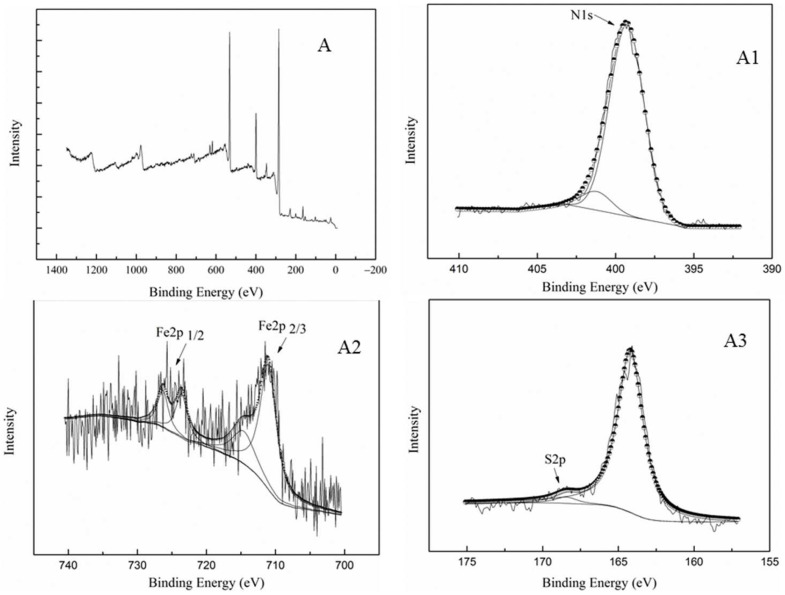
XPS spectrum of FePc@SO_2_Cl-C_lig_ ((**A**): XPS spectrum of FePc@SO_2_Cl-C_lig_; (**A1**): N 1s photopeaks; (**A2**): Fe 2p photopeaks; (**A3**): S2p photopeaks).

The surface composition of FePc@SO_2_Cl-C_lig_ was investigated using X-ray photoelectron spectroscopy (XPS), and the spectra are illustrated in Figure 5. The N1s spectrum (A1) could be split into two main peaks at 399.2 eV and 401.2 eV, which correspond to N of C-N (399.2 eV) and S-N (401.2 eV) [30,31]; for Fe2p spectrum (A2), four sets of signals could be captured in the region of 700–730 eV, which is consistent with the 2p_3/2_ orbital of Fe^2+^ (710.7 eV), 2p_3/2_ orbital of Fe^3+^ (714.6 eV), 2p_1/2_ orbital of Fe^2+^ (723.6 eV), and 2p_1/2_ orbital of Fe^3+^ (726.3 eV) [28,32,33]. Figure 5-A3 shows the spectrum of S 2p; a binding energy around 168.3 eV was observed, which is consistent with S 2p of typical sulphonamides [34,35]. It can be inferred from these data that iron phthalocyanine was successfully attached to the SO_2_Cl-C_lig_. Significantly, another sulfur signal at a binding energy around 164.2 eV corresponding to sulfoxide (–SO–) was captured, indicating that some of the sulfoxide was intact on the surface of the catalyst after acyl chlorination [36].

#### 2.1.3. BET Analysis

To check the structure and morphology, BET analyses including specific surface areas and pore volume were performed for SO_2_Cl-C_lig_ and FePc@SO_2_Cl-C_lig_.

**Figure 6:**
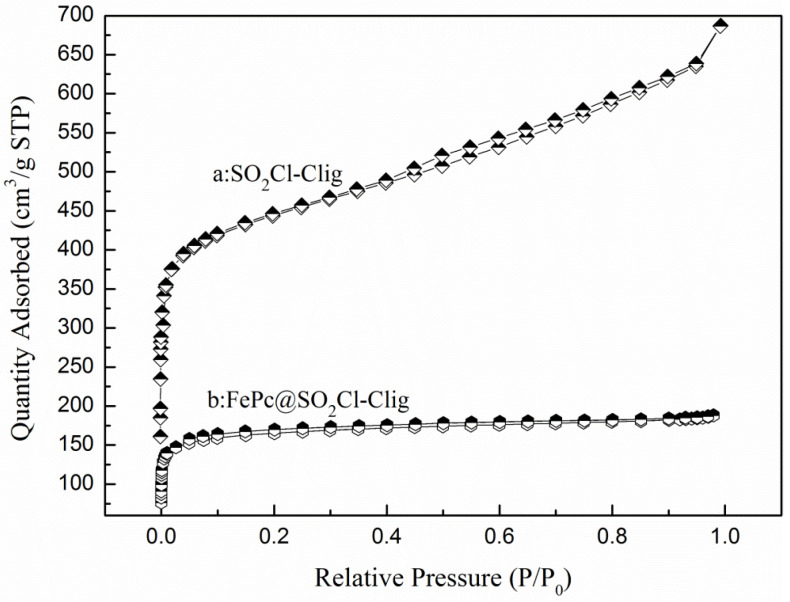
N_2_ adsorption–desorption isotherms of SO_2_Cl-C_lig_ and PcFe@SO_2_Cl-C_lig_.

**Figure 7:**
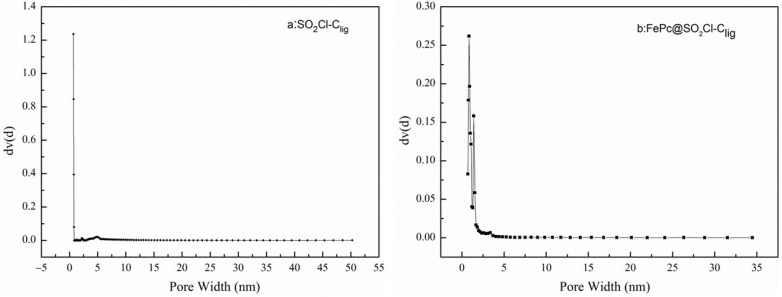
Pore size distributions of curves of (**a**) SO_2_Cl-C_lig_ and (**b**) FePc@SO_2_Cl-C_lig_.

As shown in Figure 6, a steep slope can be seen when P/P0 was below 0.2. This can confirm that the two N_2_ adsorption–desorption isotherms accord with the type I isotherm adsorption and H4-type hysteresis loop; moreover, this can also imply that a large number of micro-pores are present in the samples [37,38]. The detailed surface area and pore volume of SO_2_Cl-C_lig_ and PcFe@SO_2_Cl-C_lig_ are listed in Table 1. The sample of FePc@ SO_2_Cl-C_lig_ displayed lower surface area and pore volume, that is, the specific surface area and pore volume of two samples abide by a slight declining trend: SO_2_Cl-C_lig_ > FePc@ SO_2_Cl-C_lig_. When FePc was loaded on SO_2_Cl-C_lig_, the BET surface area decreased from 643.67 m^2^/g to 638.98 m^2^/g, while the total pore volume decreased from 0.337 cm^3^/g to 0. 291 cm^3^/g. This drop in the BET surface area and the total pore volume may be because some pores of the carrier were blocked by the FePc [39]. The pore size distribution curves of SO_2_Cl-C_lig_ and FePc@SO_2_Cl-C_lig_ are displayed in Figure 7. For SO_2_Cl-C_lig_, one strong peak and two weak peaks can be seen in the range of 0.6–2.0 nm, but regarding FePc@SO_2_Cl-C_lig_, two strong peaks can be observed in the range of 0.85–1.42 nm. The two plots confirmed the large number of micro-pores, demonstrating that after the treatment by FePc, the pore structure did not change significantly. Clearly, a peak around 4.84 nm in plot a and another peak around 3.38 nm in plot b can be observed, which belong to meso-pores. Regarding porous catalysts, micro-pores mainly provide sufficient activity sites for catalysts. Opportune meso-pores enable efficient mass transport, and the catalytic performance can be improved only if the balance is kept between active sites and mass transport [40].

#### 2.1.4. SEM Analysis

**Figure 8:**
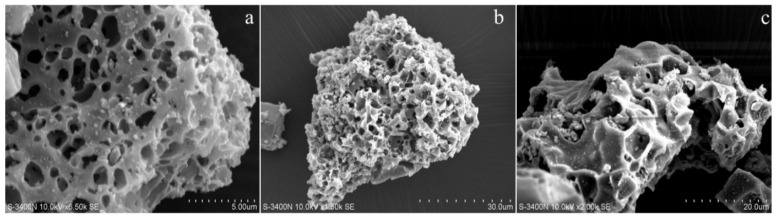
SEM images of SO_2_Cl-C_lig_ and FePc@SO_2_Cl-C_lig_ ((**a**). SO_2_Cl-C_lig_; (**b**). FePc@SO_2_Cl-C_lig_; (**c**). FePc@SO_2_Cl-C_lig_; magnified 2000 times).

SEM analysis was conducted to investigate the morphology changes in SO_2_Cl-C_lig_ and FePc@SO_2_Cl-C_lig_; the images are displayed in Figure 8.

As shown in Figure 8a, the pore structure was clearly visible and more uniformly dispersed on the surface of SO_2_Cl-C_lig_ before the loading of FePc. It can be seen obviously from Figure 8b that the sample already had abundant pores; however, when the samples were magnified 2000 times, as shown in Figure 8c, it was clearly seen that some pores were blocked by FePc, and this phenomenon confirmed the BET analysis.

### 2.2. Optimizing the Reaction Conditions

**Figure 9:**
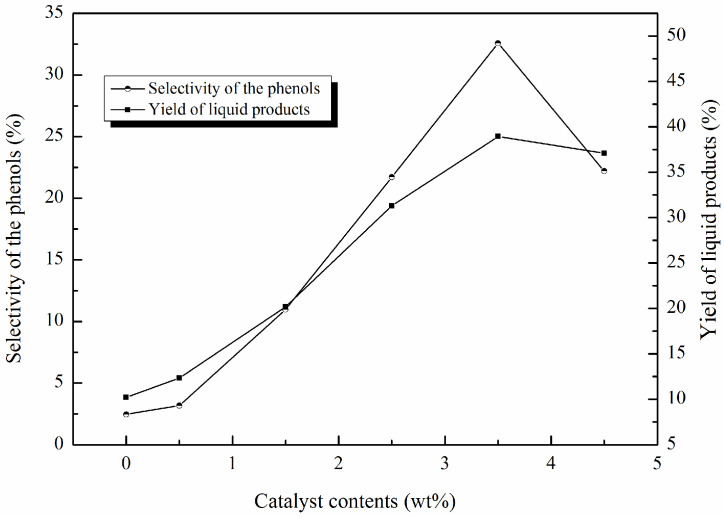
Effects of catalyst contents on the liquid product yields and the selectivity of phenolic compounds (catalyst contents: 0 wt%, 0.5 wt%, 1.5%, 2.5 wt%, 3.5 wt%, 4.5 wt%; H_2_O_2_ concentration: 15 × 10^−2^ mol/L; reaction time: 120 min; T = 135 °C pH = 3.0.).

**Figure 10:**
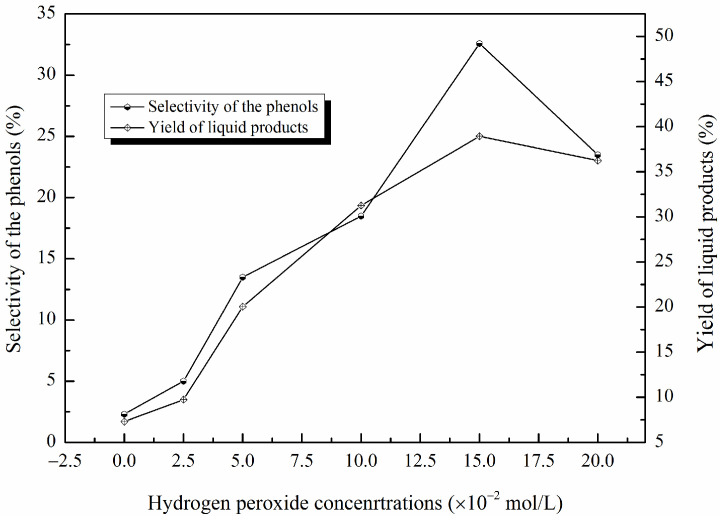
The effects of hydrogen peroxide concentrations on the yield of liquid products and the selectivity of phenolic compounds (catalyst contents: 3.5 wt%; H_2_O_2_ concentration: 0 mol/L, 2.5 × 10^−2^ mol/L, 5 × 10^−2^ mol/L, 10 × 10^−2^ mol/L, 15 × 10^−2^ mol/L, 20 × 10^−2^ mol/L; reaction time: 120 min; T = 135 °C; pH = 3.0).

**Figure 11:**
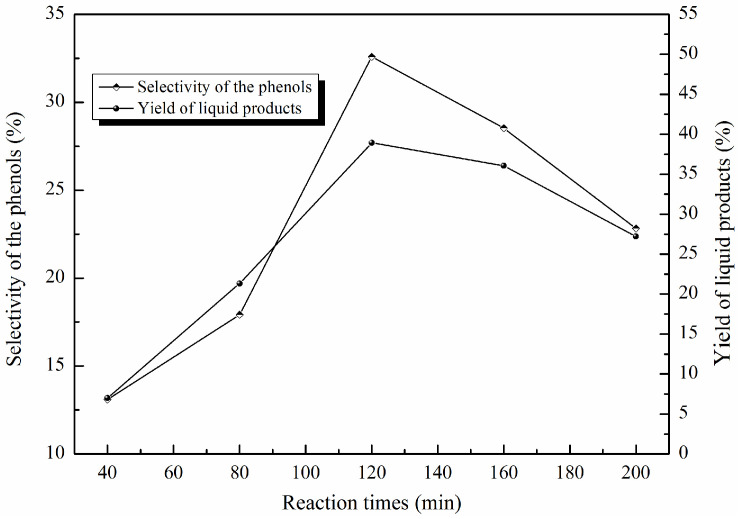
The effects of reaction time on the yield of liquid products and the selectivity of phenolic compounds (catalyst contents: 3.5 wt%; H_2_O_2_ concentration: 15 × 10^−2^ mol/L; reaction time: 40 min, 80 min, 120 min, 160 min, 200 min; T = 135 °C; pH = 3.0).

The degradation efficiency of lignin and the composition properties of liquid products were significantly affected by various reaction conditions, such as reaction temperature, reaction time, the type of catalyst and content, and the feedstock content [41]. In this study, the effects of catalyst content, reaction time, and hydrogen peroxide concentration on the degradation efficiency of lignin were investigated; the results are displayed in Figure 9, Figure 10 and Figure 11.

Figure 9 illustrates the catalyst content effects on the liquid product yields and phenol selectivity. As is known, most of the reactions were affected by the activity sites and basic properties of the catalysts. Keeping this in mind, six different catalyst concentrations were tried in this work: 0 wt%, 0.5 wt%, 1.5 wt%, 2.5 wt%, 3.5 wt%, and 4.5 wt%. It can be observed from Figure 9 that with the increase in catalyst content, the yield of liquid products and the selectivity of the phenols were both higher compared to those of the non-catalyst degradation. A maximum liquid product yield of 38.94% was observed when 3.5 wt% of catalyst was employed, while the selectivity of the phenols also reached the highest value of 32.58%. When the catalyst content increased to 4.5 wt%, the liquid product yield and the selectivity of the phenols both decreased.

These observations implied that the proper amount of the catalyst had a significant contribution to the yields of liquid products and the selectivity of the phenols. Once the amount of the catalyst increases beyond the optimum value, the yields of liquid products and the selectivity of the phenols may decrease. The catalyst not only can offer ample activity sites, but also facilitate the generation of HO· (hydroxyl) and HOO· (perhydroxyl) radicals from H_2_O_2_, and HO· can weaken the C-O or C-C linkages in lignin, subsequently resulting in the conversation of lignin into phenolic compounds and other small chemicals [42]. In this study, the maximum proportion of the phenolic compounds was found with 3.5 wt% of catalyst, suggesting that this amount of catalyst favored the generation of the amount of available OH·, and so enhanced the breaking of the C-O or C-C bonds in lignin. When the amount of catalyst exceeded 3.5 wt%, the production of more OH· contributed to the over-oxidation of the aromatic products and the coupling and condensation of the intermediates, thereby reducing the yield of liquid products and proportion of phenolic compounds [18].

Figure 10 illustrates the influence of the amount of hydrogen peroxide on the yield of liquid products and the selectivity of phenolic compounds. Six different levels (0 mol/L, 2.5 × 10^−2^ mol/L, 5 × 10^−2^ mol/L, 10 × 10^−2^ mol/L, 15 × 10^−2^ mol/L, 20 × 10^−2^ mol/L) were used with 3.5 wt% of catalyst at 135 ℃ and pH 3.0 for 120 min. Similar changing trends were observed for the yield of liquid products and the selectivity of phenolic compounds with the increase in the H_2_O_2_ concentration from 0 mol/L to 20 × 10^−2^ mol/L. The yield of liquid products and the selectivity of phenolic compounds increased as the hydrogen peroxide concentration increased from 0 mol/L to 15 × 10^−2^ mol/L. A maximum yield of 38.94% and a highest selectivity of 32.58% were obtained when 15 × 10^−2^ mol/L of hydrogen peroxide was used, further increasing the hydrogen peroxide concentration and resulting in a non-obvious change in liquid product yields, but the selectivity of phenolic compounds obviously decreased.

At a lower H_2_O_2_ concentration, the generation of HO· was not enough to induce the reaction. With the increase in the hydrogen peroxide concentrations, more HO· was generated; thus, more C-O or C-C linkages were weakened and finally cracked to form phenols or other chemicals [43,44]. However, when the hydrogen peroxide exceeded the optimum concentration, the excessive hydrogen peroxide could react with HO· to form more ${HO}_{2}^{•}$ radicals, which had a weaker activity compared to HO·. Furthermore, the concentration of HO· was reduced by the reaction with H_2_O_2_, which slowed, or even stopped, the breaking of chemical bonds in lignin (the detailed reactions are described as Formulas (1) and (2)) [16,45,46]. Therefore, the yield of liquid products and the selectivity of phenolic compounds did not increase further when 20 × 10^−2^ mol/L hydrogen peroxide was used.

H_2_O_2_ + HO → H_2_O + HOO· (1)

HOO·+ HO → H_2_O + O_2_ (2)

The yield of liquid products and the selectivity of phenolic compounds achieved at different reaction times are illustrated in Figure 11. A gradual increase was observed in the yield of liquid products and the selectivity of phenolic compounds with the increase in the reaction time to 2 h. When the reaction time was 2 h, the two values reached their maximum; with the further increase in the reaction time to 160 min and 200 min, the yield of liquid products and the selectivity of phenolic compounds declined significantly. When the reaction time was 200 min, the yield of liquid products and the selectivity of phenolic compounds exhibited their lowest values of 27.21% and 22.83%, respectively. This indicates that when the reaction time exceeded the optimal time of 2 h, it was not conducive to breaking the C-O or C-C linkage in lignin [47]. These variation trends suggest that as the reaction time increased, more HO· was generated, and the over-oxidation of the phenolic compounds, as well as the coupling and condensation of intermediates, became more pronounced [48,49].

### 2.3. Analysis of Liquid Products

#### 2.3.1. GC-MS Analysis

**Figure 12:**
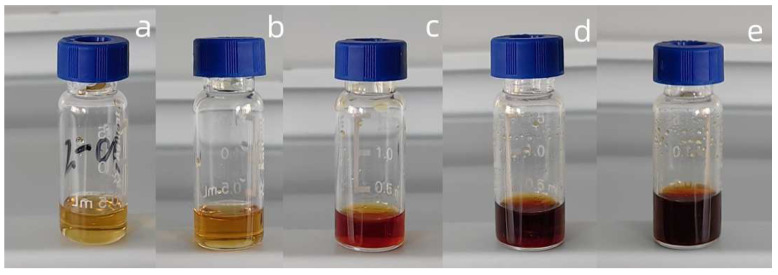
Ethyl acetate-soluble phases obtained from different catalyst contents ((**a**): 0.5 wt%, (**b**): 1.5 wt%, (**c**): 2.5 wt%, (**d**): 3.5 wt%, (**e**): 4.5 wt%. H_2_O_2_ concentration: 15 × 10^−2^ mol/L; reaction time: 120 min; T = 135 °C; pH = 3.0).

**Figure 13:**
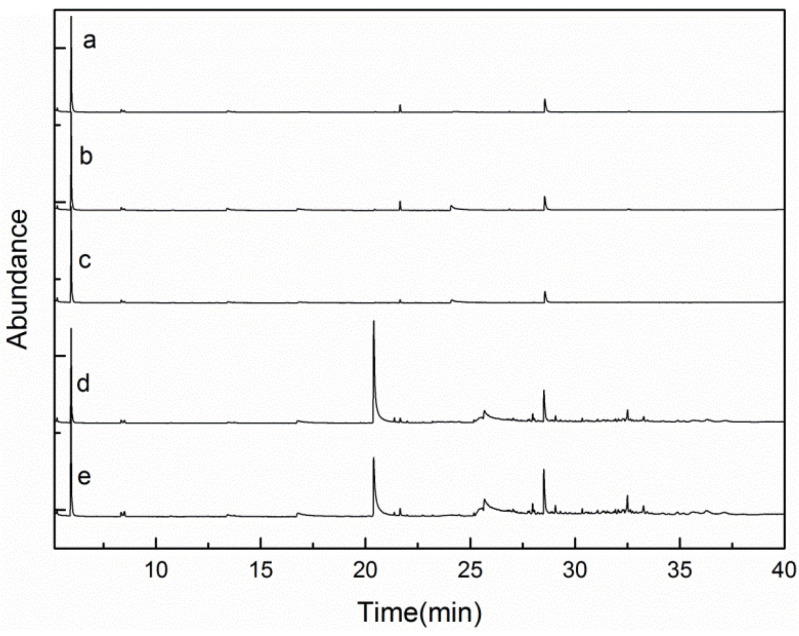
GC-MS of the depolymerization products from different catalyst concentrations ((**a**): 0.5 wt%, (**b**): 1.5 wt% (**c**): 2.5 wt%, (**d**): 3.5 wt%, (**e**): 4.5 wt%. H_2_O_2_ concentration: 15 × 10^−2^ mol/L; reaction time: 120 min; T = 135 °C; pH = 3.0).

The reaction process and the properties of liquid products were mainly examined under five different catalyst concentrations (0.5 wt%, 1.5 wt%, 2.5 wt%, 3.5 wt%, 4.5 wt%). The liquid products obtained were extracted with ethyl acetate, and the ethyl acetate-soluble aqueous phases are shown in Figure 12. The product distributions were detected via GC-MS, and the results are displayed in Figure 13.

All the samples exhibited different colors in Figure 12, and when the content of catalyst increased, the colors turned from light yellow to dark brown. From Figure 13 we can see that the degradation products obtained when using diverse catalyst concentrations had obvious differences. The products detected mainly included phenolic compounds, esters, and aldehydes, etc. The detailed information for the degradation products is summarized in Table 2.

Table 2 shows that some chemicals accounted for more of the total integral area, such as propanoic acid ethyl ester, acetic acid butyl ester, acetosyringone, phenol, 2,6-bis(1,1-dimethylethyl)- naphthalene, and 2,4-di-tert-butylphenol. Those chemicals were watched in the products obtained when different catalyst concentrations were chosen. When the catalyst concentration was 0.5 wt%, the main compound was 2,3-dihydro-benzofuran, which shared 13.72% of the total integral area. The second compound was propanoic acid ethyl ester, which accounted for 5.54% of the total integral area. Next was 2,4-di-tert-butylphenol, which made up 3.01% of the total integral area. Regarding the catalyst concentration of 1.5 wt%, the compound 2,3-dihydro-benzofuran made up most of the total integral area, that is 48.16%; this was followed by propanoic acid ethyl ester and 2,4-di-tert-butylphenol, which accounted for the total integral area of 17.42% and 10.12%, respectively. The compounds 3-hydroxy-4-methoxy-benzaldehyde and heneicosane shared 5.38% and 3.09% of the total integral area. Regarding the catalyst concentration of 2.5, 3.5, and 4.5 wt%, the compound propanoic acid ethyl ester all shared most of the total integral area, that is 65.18%, 51.59%, and 59.06%, respectively; next was 2,4-di-tert-butylphenol, which accounted for the total integral area of 15.73%, 14.84%, and 17.08%, respectively.

**Figure 14:**
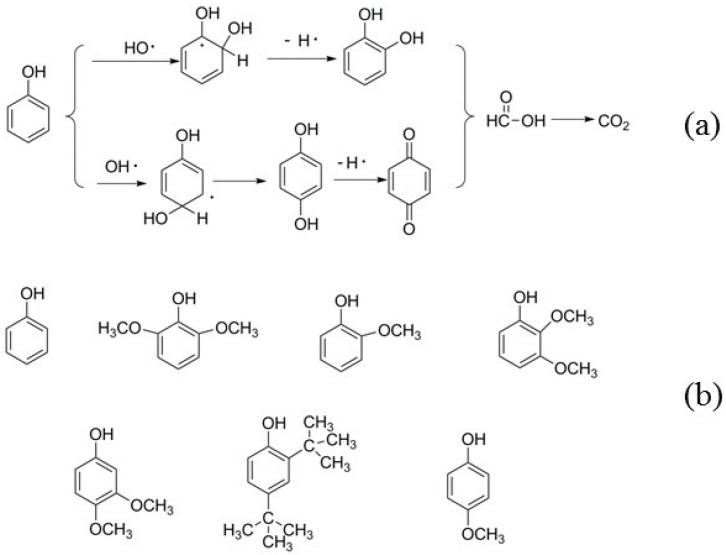
The over-oxidation of the phenols (**a**) and summary of the phenolic compounds (**b**).

It is worth noting that as the catalyst concentration increased to 3.5 wt%, the total proportion of the phenolic compounds reached a maximum. The kind of phenolic compound also changed, and the obtained phenolic compounds included phenol, 2-methoxy-phenol, 2,4-di-tert-butylphenol, 2,3-dimethoxyphenol, 3,4-dimethoxy-phenol, and 2,6-dimethoxy-phenol. 2,4-di-tert-butylphenol accounted for the maximum proportion of total integral area, that is, 14.84%; 2,6-dimethoxy-phenol accounted for 10.78% of the total integral area. The compounds 2,3-dimethoxyphenol and 3,4-dimethoxy-phenol accounted for relatively less of the total integral area, and could only be identified in the case of 3.5 wt% catalyst treatment. Regarding 2,4-di-tert-butylphenol, with the increase in the catalyst content from 0.5 wt% to 4.5%, its proportion increased from 3.01% to 17.08%, indicating that this catalyst encouraged the production of 2,4-di-tert-butylphenol. Regarding phenol, with the increase in the catalyst content from 0.5 wt% to 4.5%, its proportion increased from 0.16% to 4.22% first and then declined to 3.7% when 4.5 wt% of catalyst was used. When 0.5 wt% and 1.5 wt% of catalyst was used, there was no 2,6-dimethoxy-phenol, and when the amount of catalyst increased from 2.5 wt% and 3.5 wt%, the proportion of 2,6-dimethoxy-phenol increased to 2.15% and 10.78%, respectively. However, when the catalyst concentration further increased to 4.5 wt%, the proportion of 2,6-dimethoxy-phenol declined to 0.9%. The proportions of 0.61%, 0.72%, and 1.56% for 2-methoxy-phenol could be observed when the catalyst was 1.5 wt%, 2.5 wt%, and 3.5 wt%, respectively. When the catalyst concentration was further increased to 4.5%, its proportion decreased to 0.61%. As discussed above, the greater catalyst content can accelerate more HO· generated from H_2_O_2_, and the excessive HO· can result in the over-oxidation of the phenols (Figure 14a, taking phenol for instance) [49], thereby increasing the proportion of the phenols reduced when the catalyst concentration further increased. The identified aromatic monomers are summarized in Figure 14b.

#### 2.3.2. FT-IR and ^1^H-NMR Analysis

**Figure 15:**
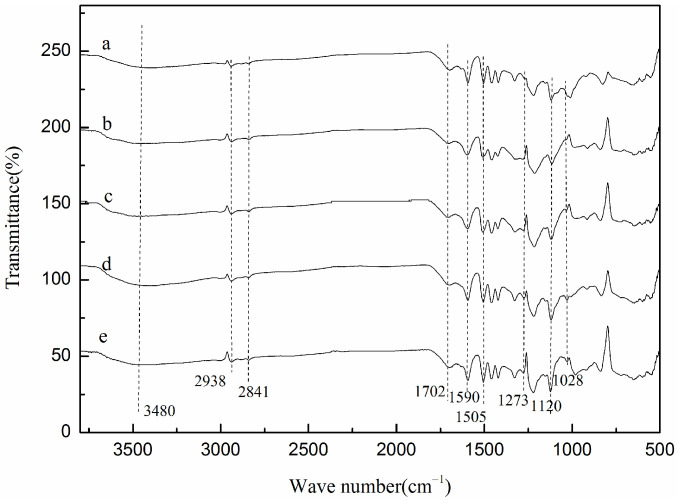
FTIR spectra of products obtained from different catalyst contents (catalyst contents: (**a**): 0.5 wt%, (**b**): 1.5 wt% (**c**): 2.5 wt%, (**d**): 3.5 wt%, (**e**): 4.5 wt%. H_2_O_2_ concentration: 15 × 10^−2^ mol/L; reaction time: 120 min; T = 135 °C; pH = 3.0).

**Figure 16:**
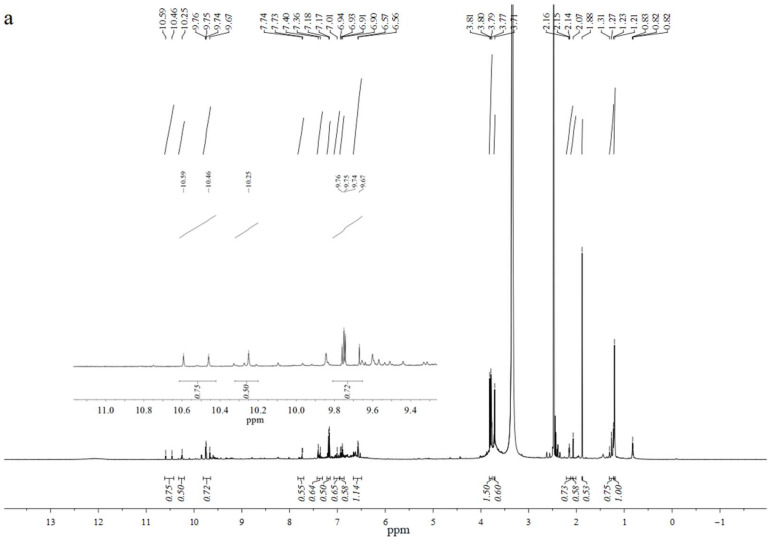

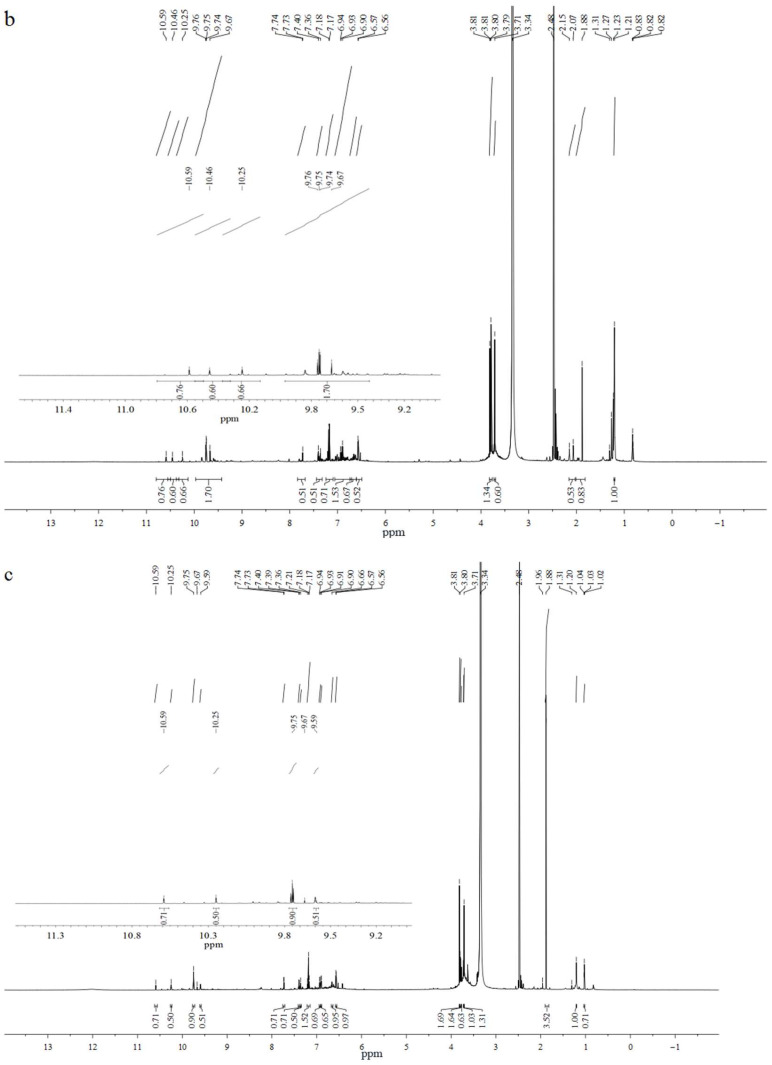

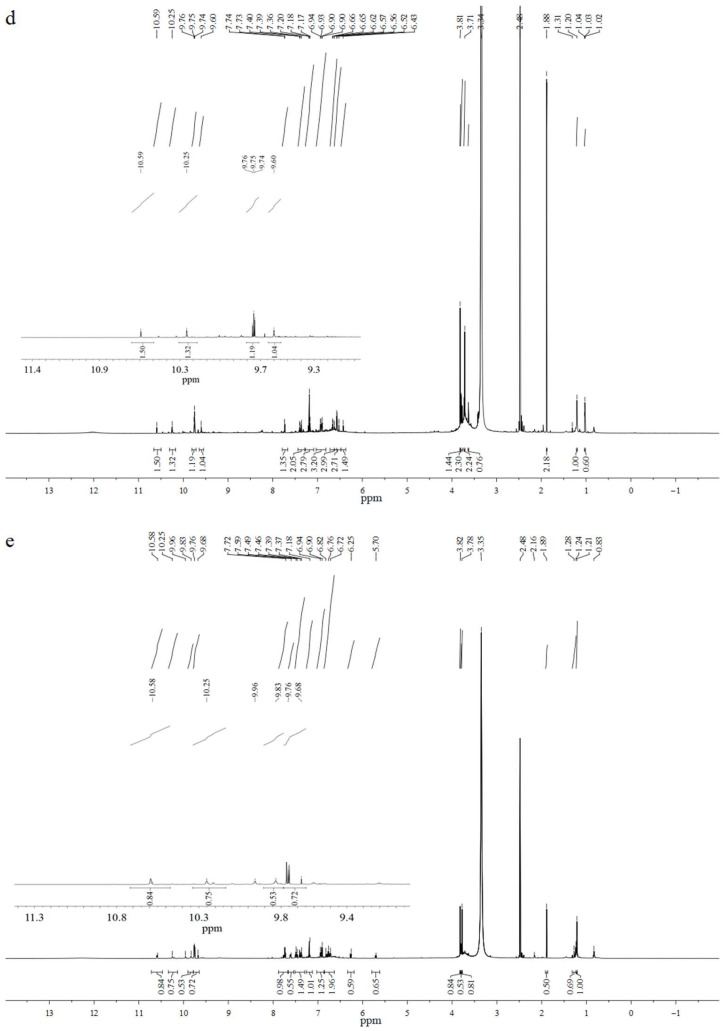
^1^H-NMR spectra of ethyl acetate-soluble products (catalyst contents: (**a**): 0.5 wt%, (**b**): 1.5 wt% (**c**): 2.5 wt%, (**d**): 3.5 wt%, (**e**): 4.5 wt%. reaction time: 120 min; H_2_O_2_ concentration: 15 × 10^−2^ mol/L; T = 135 °C; pH = 3.0).

In order to further confirm the components of the degradation products, FT-IR analysis and ^1^H-NMR analysis were conducted; the spectra are displayed in Figure 15 and Figure 16, respectively.

As shown in Figure 15, the products had relatively similar chemical groups, and the position of each vibrational peak was basically the same. These results imply that the main composition categories of the products were alike. The wide band at 3480 cm^−1^ was assigned to the combination and overlap of the stretching vibrations of O-H, =C-H, and N–H in aromatic or aliphatic groups, implying the presence of phenols or benzene in the products. At 2938 and 2841 cm^−1^, the weak vibration peaks of aliphatic C-H were generated by the asymmetric stretching vibration of C-H and symmetric stretching vibration of methyl and methylene, respectively, indicating the formation of saturated aliphatic hydrocarbons from lignin depolymerization [50]. The small and broad bands around 1702 cm^−1^ were due to the stretching vibration of C=O from ketone or carboxylic acid derivatives. The peaks around 1590 and 1505 cm^−1^ were assigned to the vibrations of C=C bonds in the aromatic skeleton. These results showed that the aromatic skeleton structure of lignin was well retained in the products [51], and these aromatic skeletons mainly existed in the form of phenols or phenol derivatives. The signals appearing at 1273 and 1120 cm^−1^ were regarded as Ar-O linkages of methoxy groups containing the phenols. Ester groups were confirmed by the peak at 1028 cm^−1^ [52].

However, some differences could be found in products obtained with different quantities of catalysts: (1) The intensity of the peak at 3480 cm^−1^ was relatively higher for the products obtained with 3.5 wt% catalysts, suggesting that, under this condition, more β-O-4 linkages were cracked to yield phenolic compounds. (2) The intensity of the peaks at 1273, 1120, and 1028 cm^−1^ was also higher in products with 3.5 wt% catalyst compared to that in the products with 0.5 wt% to 2.5 wt% catalysts, verifying the above analysis results. (3) The intensity of the peaks at 1273, 1120, and 1028 cm^−1^ in products with 3.5 wt% catalysts was somewhat similar to that in the products with 4.5 wt% catalysts, according to GC-MS analysis. With the increase in catalyst content from 3.5 wt% to 4.5 wt%, the proportion of the phenolic compounds only dropped slightly from 32.58% to 22.29%. As a result, a similar FT-IR spectrum was gained. (3) The intensity of the peaks at 1028 cm^−1^ enhanced gradually in the products using 0.5 wt% to 2.5 wt% catalyst, and was finally similar to that of the products with 3.5 wt% and 4.5 wt% catalyst. According to the GC-MS results, when 0.5 wt% and 1.5 wt% catalyst were used, the proportion of major ester compounds was 5.54% and 17.64%, respectively; when the amount of catalyst increased to 2.5 wt%, 3.5 wt%, or 4.5 wt%, the proportion of major ester compounds increased to about three or four times. This well explained the FT-IR changes for the intensity of the peaks at 1028 cm^−1^.

Clearly, the proton signals were mainly distributed in four regions: δ0.8–1.9 ppm, δ3.3–4.0 ppm, δ6.0–8.0 ppm, and δ9.5–11 ppm. The peaks occurring at δ0.8–1.9 ppm were assigned to methyl and methylene mainly from the aliphatic chain. The next strong signal, appearing at δ3.3–4.0 ppm, was attributed to the protons from methoxy [47,53]. It is obvious that a relatively high proportion of methoxy protons could be captured with 3.5 wt% catalyst. On the one hand, this phenomenon confirms the presence of a high area of methoxy chemicals such as 2,6-dimethoxy-phenol, 2-methoxy- phenol, and acetosyringone. On the other hand, the presence of the compounds 2,6-dimethoxy-phenol and 2-methoxy- phenol can concurrently prove the breaking of guaiacol and syringol sub-aromatic units of lignin under the action of the catalyst. The presence of aromatic protons was verified by the signals between the regions of 6.0 and 8.0 ppm [54], which exhibited the presence of the aromatic compounds such as phenol, 2-methoxy-phenol, acetosyringone, 2,6-dimethoxy-phenol, and 2,4-di-tert-butylphenol. The relatively high proportion of aromatic protons was observed in the case of 3.5 wt% catalyst, which is in agreement with the GC-MS results. The weak peaks observed at δ9.5–11 ppm were attributed to the protons form aldehyde or acidic groups, which validated the GC-MS analysis of the emergence of formic acid, 3,4-dimethyl-benzaldehyde, etc.

#### 2.3.3. Elemental Analysis

The elemental compositions in the liquid products were investigated and the results are shown in Table 3.

The elemental analysis results showed that the content of carbon increased in liquid products compared to that in lignin. The highest carbon content was obtained when 3.5 wt% catalyst was used, and the hydrogen content increased slightly compared to that in lignin. The oxygen content decreased compared to that in lignin, and the lowest oxygen content of 21.8% was found in the products with 3.5 wt% catalyst. This result implies that the deoxygenation or decarbonylation were improved by the catalyst [54]. Additionally, the ‘higher heating value’ (HHV) was enhanced in products, with lignin showing HHV of 20.5 MJ/kg. The value of HHV increased to 23.3–30.2 MJ/kg in the products and the highest HHV of 30.2 MJ/kg was found in the products with 3.5 wt% catalyst. As is known, the higher content of carbon can result in a higher HHV value [55].

### 2.4. Characterization of the Solid Residue Fractions

#### 2.4.1. FT-IR Analysis

**Figure 17:**
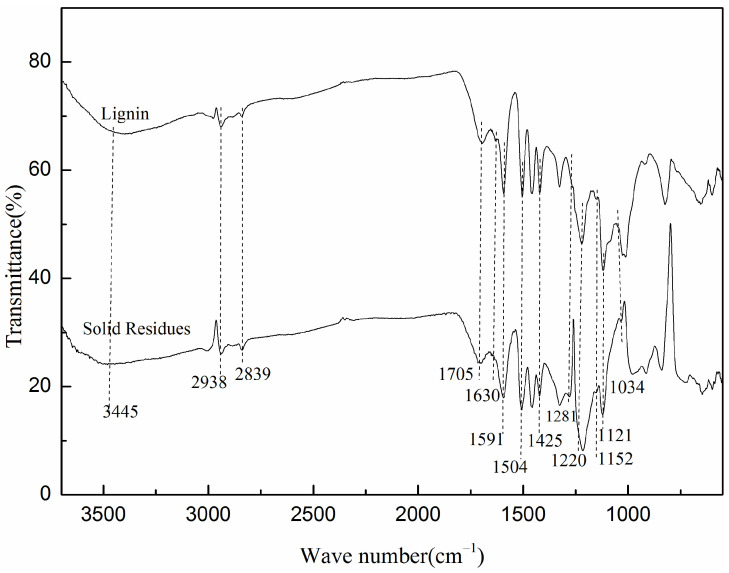
FT-IR spectrum of lignin and solid residues obtained when 3.5 wt% catalyst was used (reaction time:120 min; H_2_O_2_ concentration = 15 × 10^−2^ mol/L; T = 135 °C; pH = 3.0).

To investigate the changes in the chemical structures of lignin before and after the FePc@SO_2_Cl-C_lig_ treatment, FT-IR analyses of lignin and residues were performed and the results are depicted in Figure 17.

The peaks occurring around 3400–3500 cm^−1^ were assigned to O-H stretching vibration. The weak peaks at 2938 cm^−1^ and 2839 cm^−1^ issued from C-H stretching vibrations in methyl and methylene groups [56]; the signals around 1705 cm^−1^ were attributed to the ester groups; and the weak peak at 1630 cm^−1^ corresponded to C=O stretching of carbonyl. The bands at 1591, 1504, and 1425 cm^−1^ were attributed to the skeletal vibrations of the benzene ring, and the peaks appearing at 1121 cm^−1^ and 1152 cm^−1^ corresponded to the C-O-C stretching vibration [57];

As is well known, the solid residues were mainly generated from the decomposition of the lignin and polycondensation of the intermediates. In comparison, some dramatic changes can be discovered for the solid residues: (1): The intensity in the region corresponding to the skeletal vibrations of the benzene ring slightly declined. The presence of this region pointed to the fact that the solid residues retained the core structure of lignin; furthermore, the weaker intensity of these peaks indicated the degradation of lignin after FePc@SO_2_Cl-C_lig_ treatment. (2) The weak peak observed in lignin at 1630 cm^−1^, corresponding to C=O stretching of carbonyl, disappeared in solid residues, suggesting that the de-carbonylation reaction occurred during the degradation of lignin. (3) The typical signals for C-O-C stretching vibrations at 1121 cm^−1^ and 1152 cm^−1^ became weaker, respectively, indicating that the β-O-4 ether bond cleaved during the degradation process under the action of FePc@SO_2_Cl-C_lig_. (4) The band at 1220 cm^−1^ in lignin shifted to a lower wavenumber of 1216 cm^−1^ in the solid residues, indicating some new structures generated during the degradation process. (5) Some new peaks were observed in solid residues at 1281 cm^−1^ and 1034 cm^−1^, which also indicated that the new structures were formed; this was because, during degradation, the small intermediate molecules reacted with the chains of lignin and generated new chain structures [58].

#### 2.4.2. ^1^H-NMR Analysis

**Figure 18:**
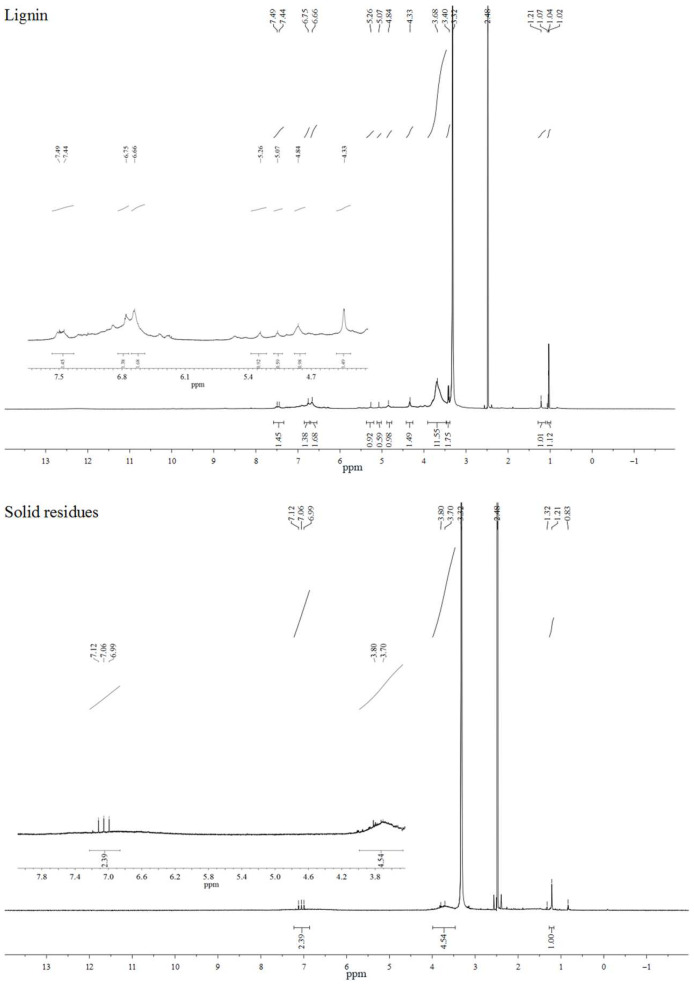
^1^H-NMR spectrum of lignin and solid residues obtained when 3.5 wt% catalyst was used (reaction time:120 min; H_2_O_2_ concentration = 15 × 10^−2^ mol/L; T = 135 °C; pH = 3.0).

In order to gain further information about the structure changes of lignin and solid residues, ^1^H-NMR analysis was conducted, and the results are displayed in Figure 18.

As can be seen from Figure 18, lignin exhibited strong peaks as compared to the solid residues. For lignin, the strong signals mainly occurred at four regions: the signals corresponding to methylene groups of the aliphatic chain were observed at the region from 1 to 1.3 ppm; the methoxyl group signals occurred around 3.7 ppm; the signals appearing at 4–5.3 ppm were assigned to H-α and H-β in β-O-4 substructures; and the typical signals corresponding to aromatic protons were observed in their regions of 6.5–7.5 ppm [59,60]. However, for solid residues, the signals for methoxyl groups around 3.7 ppm and the typical signals corresponding to aromatic protons between 6.5 and 7.5 ppm diminished significantly. Furthermore, the signals assigned to β-O-4 substructures almost disappeared compared with lignin. The findings further confirmed the FT-IR results, also indicating that the breaking of β-O-4 linkages was improved by FePc@SO_2_Cl-C_lig_.

#### 2.4.3. GPC Analysis

To investigate if the degradation of lignin increased under the treatment of FePc@SO_2_Cl-C_lig_, the molecular weights of lignin and solid residues obtained at different reaction times (40 min, 80 min, 120 min, 150 min) were measured with the assistance of GPC analysis. The reported data including weight-average (Mw) and number-average (Mn) molecular weights and polydispersityare (Mw/Mn) of lignin and solid residues, which are listed in Table 4.

During the degradation process of lignin, small molecule chemicals, superpolymers, or char could all be formed by a range of reactions. In this process, the degradation of lignin and the polycondensation of intermediates take place simultaneously and compete with each other. The cleavage of β-O-4 results in the reduction in the molecular weight; conversely, the formation of superpolymers or char may result in an increase in the molecular size [61]. As can be seen from Table 4, lignin had an Mw of 66,671 g/mol and the polydispersityare value was 1.81. In comparison, a distinct reduction in the molecular weight for solid residues was observed; the value of Mw fell by almost half for the solid residues obtained at 80 min and 120 min. However, the molecular weight showed a slight increase to 33,468 g/mol for the solid residues obtained with 150 min reaction time. This can be explained because the polycondensation gradually became the major reaction at this stage. At the same time, the polydispersity index of solid residues declined to 1.51 and 1.22 when the reaction time was 80 min and 120 min, demonstrating that the molecular weight distributions of solid residues were relatively narrow at this two stage. The index then increased to 2.29 with the extension in reaction time to 150 min. This phenomenon also confirmed that the polycondensation reaction was dominant at this stage. In addition, the low polydispersity index values (<3) manifested that lignin molecularly belongs to a kind of uniform-sized polymer [62]. All these changes validate that the lignin was subjected to the cleavage of the ether bonds when using FePc@SO_2_Cl-C_lig_ as a catalyst.

#### 2.4.4. SEM Analysis

**Figure 19:**
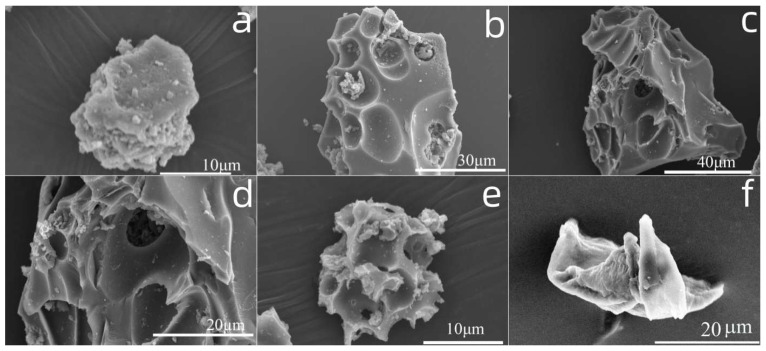
SEM images of solid residues at different reaction times (conditions: 3.5 wt% of catalyst, T = 135 °C, concentration of H_2_O_2_: 15 × 10^−2^ mol/L; pH = 3.0. (**a**): lignin, (**b**): residues at 40 min, (**c**): residues at 80 min, (**d**): enlargement of residues at 80 min, (**e**): residues at 120 min, (**f**): residues at 150 min).

SEM analysis was performed to investigate the native microstructure alteration of lignin during the degradation process at different reaction times (40 min, 80 min, 120 min, 150 min); the results are depicted in Figure 19.

As can be seen from Figure 19a, the original lignin had a relatively smooth and tight surface structure. When the reaction time was increased to 40 min (Figure 19b), the smooth surface was slightly destroyed; some shallow pits were observed and a small amount of dross was deposited on the surface. This observation indicated that the degradation of lignin begins on the surface. At the same time, this GPC analysis result made it clear that when the reaction time was 40 min, the Mw value only fell slightly to 62,256 g/mol; with a further increase in reaction time to 80 min (Figure 19c), the surface damage intensified and some deeper pits arose. Magnification of this figure (Figure 19d) shows that more fragments attached on the surface or in the pits. This phenomenon demonstrated that at this stage the main reaction was depolymerization. When the reaction time continued to increase to 120 min (Figure 19e), slight caking could be captured. This implies that the coupling and polycondensation reaction gradually began to become obvious with the increase in the reaction time. When the reaction time was further increased to 150 min (Figure 19f), conglomerations could be observed. The longer time promoted the coupling and polycondensation to a much greater extent. Combined with the GPC analysis, this also explained why the weight average of solid residues increased after the reaction time of 2 h.

## 3. Experimental

### 3.1. Materials

The key experiment reagents are listed as follows:

Lignin (prepared in the lab); iron chloride hexahydrate (FeCl_3_·6H_2_O), hydrogen peroxide (H_2_O_2_), thionyl chloride (SOCl_2_), 4-nitrophthalic acid (C_8_H_5_NO_6_), urea (CO(NH_2_)_2_), sodium sulfide (NaS_2_·9H_2_O), and *N*,*N*-dimethylformamide (C_3_H_7_NO) were provided by Aladdin. Potassium hydroxide (KOH, purity of ≥98%), sodium hydroxide (NaOH, purity of ≥97%), sulfuric acid (H_2_SO_4_, concentration of 98%), and ethanol (C_2_H_5_OH, 99.9%) were provided by JiKun Chemicals Company (Nanning, China).

### 3.2. Experimental Procedure

#### 3.2.1. Catalyst Preparation

##### Manufacture of Sulphonyl Chloride Derivative of Lignin-Base Carbon

**Figure 20:**
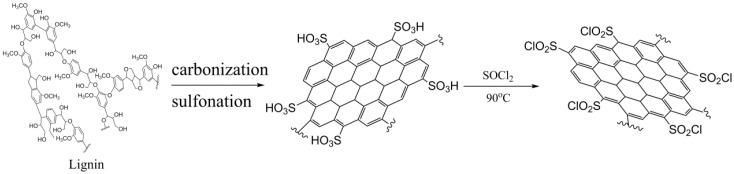
The synthesis route of SO_2_Cl-C_lig_.

Lignin used in this work was refined from black pulping liquor with H_2_SO_4_ (concentration: 65%) and then dried at a constant temperature of 105 °C for 12 h. The sulphonyl chloride derivative of lignin-base carbon was synthesized via three steps: carbonization, sulfonation, and acyl chlorination. First, carbonization of lignin was conducted in a tube furnace under the protection of N_2_. Lignin and KOH were placed in a nickel disc in proportion and then placed in a tube furnace; the tube furnace was heated to 550 °C with the ramp rate of 15 °C/min and finally maintained for 90 min. After the reaction, the obtained black sample (named C_lig_) was washed and filtered with hot water, then dried at 105 °C for 12 h. Second, sulfonation of C_lig_ was conducted in a three-necked round-bottom flask under the protection of N_2_. C_lig_ and H_2_SO_4_ were mixed in the round-bottom flask in proportion, and the reaction was kept for 6 h at 150 °C. The final black sample (named SO_3_H-C_lig_) was washed and filtered with water, and finally dried at 105 °C for 12 h. Third, the sulphonyl chloride derivative was synthesized using the method in a previous report [63]; SO_3_H-C_lig_ and an overabundance of SOCl_2_ were added to the mixed solvents of *N*,*N*′-dimethylformamide (DMF) and water. The reaction was performed at 90 °C for 24 h; then the residual SOCl_2_ and solvent were removed by distillation. The solid products (named SO_2_Cl-C_lig_) were washed and dried for the next application. The synthesis method of SO_2_Cl-C_lig_ is described in Figure 20.

##### The Synthesis of Iron Amino Phthalocyanine (FePc-NH_2_)

**Figure 21:**
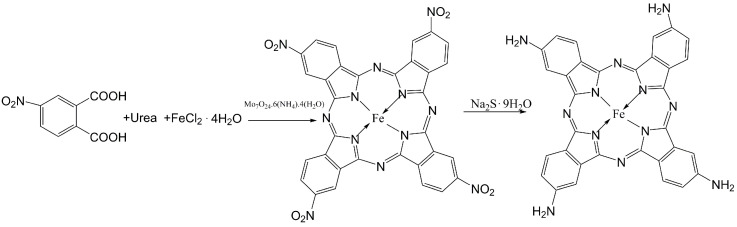
The synthesis route of iron amino phthalocyanine.

Iron amino phthalocyanine was synthesized through two steps: first, iron tetranitro phthalocyanine was prepared according to a previous method [64]; second, the prepared iron tetranitro phthalocyanine was ammoniated by the reaction with sodium sulfide nonahydrate (NaS·9H_2_O) at 65 °C for 4 h to finally yield the objective product [65]. In this reaction, the molar ratio of iron tetranitro phthalocyanine and NaS·9H_2_O was 1:15. The final reaction mixture was treated with 0.5 M HCl and 1.0 M NaOH. The obtained blue-green product (named FePc-NH_2_) was washed with water and ethanol, and dried at 65 °C for 12 h. The detailed synthesis route of FePc-NH_2_ is described in Figure 21.

##### Synthesis of Sulfonyl-Chloride-Modified Lignin-Based Porous Carbon-Supported Iron Phthalocyanine

**Figure 22:**
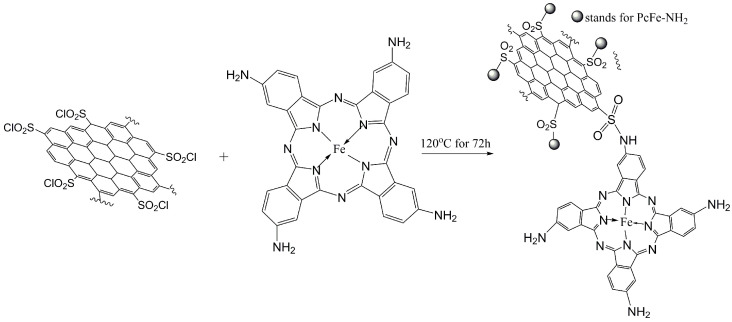
The synthesis route of FePc@SO_2_Cl-C_lig_.

The above prepared SO_2_Cl-C_lig_ and FePc-NH_2_ were added to a mixture of DMF and H_2_O and sonicated for 2 h; then, the mixture was stirred for 72 h at 120 °C [30]. Finally, the reaction mixture was filtered off, and the solid residues were washed with water and acetone, and dried at 65 °C to yield a black-blue powder (named FePc@SO_2_Cl-C_lig_). The synthesis route of FePc@SO_2_Cl-C_lig_ is displayed in Figure 22.

#### 3.2.2. Treatment of Lignin with FePc@ SO_2_Cl-C_lig_

The final experiment was performed in a 150 mL reactor. First, the appropriate amount of FePc@SO_2_Cl-Clig catalyst (catalyst-to-lignin ratio from 0 wt% to 4.5% wt%) was dispersed in a combined solvent of water and acetonitrile; second, the appropriate amount of lignin (1 g) was added to the above mixture, and the pH of the mixture was adjusted to the designed value (pH = 3.0 in this work) with sulfuric acid. Subsequently, the mixture was vibrated for 20–30 min to fully disperse the FePc@SO_2_Cl-C_lig_ and lignin. Finally, the mixture was transferred into the reactor; simultaneously, H_2_O_2_ (from 0 mol/L to 20 × 10^−2^ mol/L) was added to the reaction mixture and the mixture was stirred at 135 °C for 40–200 min. After the reaction finished, the solid residues and liquid products were gained by centrifugal separation; the liquid products were extracted by ethyl acetate and then used for analysis.

### 3.3. Characterizations

The type of chemical functional group of the catalyst was recorded using Fourier transform infrared spectroscopy (FTIR) on Nicolet IS10 (Waltham, MA, USA). Element compositions and speciation were detected by X-ray photoelectron spectroscopy (XPS) on a thermo EscaLab 250 Xi (Norristown, PA, USA). The BET surface area was tested using N_2_ adsorption/desorption isotherms (Quantachrome autosorb IQ-C analyzer, Boynton Beach, FL, USA). The surface structure of catalysts was recorded using a Hitachi s-3400 Scanning Electron Microscope (Hitachi, Tokyo, Japan).

Chemical distributions of degradation products were investigated via GC/MS chromatograms using an Agilent (Santa Clara, CA, USA) 6890A/5973N series instrument fitted with a HP-5MS (30 m × 0.25 mm × 0.25 um) column. A quantity of 2 µL of sample was injected into the system at 40 °C. The column was maintained at 40 °C for 2 min; the first ramp was for 5 °C/min to 150 °C and maintained for 2 min, followed by a ramp for 8 °C/min to 280 °C, which was held for 5 min. Chemical structures and distributions of degradation products were characterized by ^1^H-NMR determinations on an Agilent DD2 800 MHz NMR spectrometer (Santa Clara, CA, USA)with deuterated dimethyl sulfoxide (Dimethyl sulfoxide-d6) as the standard solvent, and FTIR on a Nicolet IS10 (Waltham, MA, USA). Element analysis (EA) was used to analyze the element compositions of the products.

The solid residues were characterized via FTIR on a Nicolet IS10 (Waltham, MA, USA). ^1^H-NMR determinations were carried out on an Agilent DD2 800 MHz NMR spectrometer (Santa Clara, CA, USA) with deuterated dimethyl sulfoxide (Dimethyl sulfoxide-d6) as the standard solvent. GPC analysis was conducted using a 1260 Infinity II GPC/MDS instrument (Santa Clara, CA, USA) with DMF as mobile phase, and SEM analysis was conducted on a Hitachi s-3400 Scanning Electron Microscope (Hitachi, Tokyo, Japan).

The liquid product yield was calculated as follows:

liquid product (wt%) = W_liquid_/W_lignin_ × 100% (3)

where W_lignin_ and W_liquid_ represent the weight of lignin and the weight of liquid products, respectively.

The selectivity of the phenols was presented as the ratio of the peak area of the corresponding products to the total area of the liquid products.

## 4. Conclusions

In this study, sulfonyl-chloride-modified lignin-based porous carbon-supported metal phthalocyanine catalyst with a high surface area of 638.98 m^2^/g and a pore volume of 0.291 cm^3^/g was manufactured and employed for lignin degradation via Fenton reaction. The catalyst exhibited good ester and phenol selectivity according to GC-MS analysis. The highest liquid product yield of 38.94% and the highest phenol selectivity of 32.58% were observed at 135 ℃ with 3.5 wt% of catalyst and 15 × 10^−2^ mol/L H_2_O_2_ for 120 min. The main phenols were phenol, 2-methoxy-phenol, 2,4-di-tert-butylphenol, 2,3-dimethoxyphenol, 3,4-dimethoxy-phenol, and 2,6-dimethoxy-phenol. The analysis results for liquid products showed that (1) the composition of liquid products was different under various catalyst concentrations; and (2) the highest calorific value of 30.2 MJ/kg was acquired with 3.5 wt% catalyst. The analysis results for solid residues showed that (1) the M_W_ of residues was almost half that of the lignin; and (2) the degradation of the lignin started on the surface. These findings offer a significant theoretical reference for lignin degradation using a lignin-based porous carbon-supported metal phthalocyanine catalyst via Fenton reaction.

## Figures and Tables

**Table 1:** Characterization of the porous texture of SO_2_Cl-C_lig_ and PcFe@SO_2_Cl-C_lig_.

Samples	S_BET_ (m^2^/g)	S_Mic_ (m^2^/g)	V_total_ (cm^3^/g)	V_Mic_ (cm^3^/g)
SO_2_Cl-C_lig_	643.67	540.79	0.337	0.210
FePc@ SO_2_Cl-C_lig_	638.98	603.57	0.291	0.246

**Table 2:** GC-MS analysis results for bio-oils using different catalyst contents.

Retention Time/s	Compound Name	Catalyst Concentrations (wt%) Quantitative Content (Area %)				
		0.5	1.5	2.5	3.5	4.5
5.712	Propanoic acid, 3-mercapto-, methyl ester	-	0.14	-	-	0.35
5.958	Propanoic acid, ethyl ester	5.54	17.42	65.18	51.59	59.06
8.344	Acetic acid, butyl ester	0.33	0.92	3.28	2.65	2.92
8.489	Acetosyringone	0.82	0.84	1.49	1.38	1.88
10.753	Cyclohexanone	-	0.17	0.11	0.08	-
12.449	2-Anthracenamine	0.08	-	-	-	-
13.431	Phenol	0.16	0.24	3.11	4.22	3.70
13.734	1,2,4-trimethoxybenzene	-	0.14	0.46	0.41	0.49
15.611	Eicosyl nonyl ether	-	0.09	-	-	-
16.818	Phenol, 2-methoxy-	-	0.61	0.72	1.56	0.61
16.923	Mequinol	0.13	-	-	-	-
18.42	formic acid	-	-	0.16	0.10	0.14
19.095	2-Isopropylpyrazine	-	-	0.08	-	-
19.765	Dodecane	-	0.13	-	-	-
20.154	Formic Acid, 4-methoxyphenyl ester	-	0.08	-	-	-
20.408	Benzofuran, 2,3-dihydro-	13.72	48.16	-	-	-
20.44	Benzaldehyde, 3,4-dimethyl-	-	-	0.74	1.23	0.24
21.202	Benzonitrile, 2-(4-methylphenyl)-	-	-	-	-	0.11
21.375	Benzene, 1,3-bis(1,1-dimethylethyl)-	0.16	0.94	0.27	0.16	0.40
21.655	Naphthalene, 2,6-bis(1,1-dimethylethyl)-	0.27	1.05	2.56	5.78	5.41
21.985	(S)-(−)-2-Amino-3-phenyl-1 -propanol	-	-	-	0.09	-
21.991	Dodecane, 1-iodo-	-	0.40	-	-	0.11
22.004	1,4-Benzenediamine, *N*,*N*′-bis(1-methylethyl)-	0.18	-	-	-	-
22.363	Octane, 2,3,6,7-tetramethyl-	-	0.07	-	-	-
22.666	Ethanone, 2-(2-benzothiazolylthio)-1-(3,5-dimethylpyrazolyl)-	-	-	-	-	0.09
22.737	Acetaldehyde, bis(1-methylethyl)hydrazone	0.11	0.27	-	-	-
22.809	Benzaldehyde, 2,4,5-trimethyl-	-	-	-	-	0.07
23.198	Linoleic acid, ethyl ester	-	-	0.18	0.17	-
23.204	Undecanoic acid		-	-	-	0.18
23.21	Hexadecane, 1-iodo-	-		-	--	-
24.119	Phenol, 2,6-dimethoxy-	-	-	2.15	10.78	0.90
25.186	Tetradecane	0.12	0.68	-	-	-
25.556	[1,2,4]Oxadiazole, 5-(4-tert-butylphenoxymethyl)-3-(thiophen-2-yl)-	-	-	-	-	0.224
25.632	Benzaldehyde, 4-hydroxy-	0.29	-	-	-	-
25.798	Benzaldehyde, 3-hydroxy-4-methoxy-	0.47	5.38	-	-	-
26.872	desaspinidol	-	-	0.52	0.84	0.69
27.049	3-Ethyl-2,6,10- trimethylundecane	-	0.33	-	-	-
27.072	Dimethyl phthalate	-	-	0.34	0.20	0.12
27.472	2-Heptanone, 3-propylidene-	-	-	-	0.12	-
27.753	Tetradecane, 2,6,10-trimethyl-	-	0.25	-	-	-
27.982	Octacosane, 1-iodo-	0.362	1.62	-	-	-
27.993	Hexacosane	-	-	-	-	0.18
27.999	2,3-Dimethoxyphenol	-	-	-	0.08	-
28.056	Phenol, 3,4-dimethoxy-	-	-	-	0.18	-
28.062	Pentadecane	-	0.69	-	-	-
28.073	Nonahexacontanoic acid	-	-	-	-	0.20
28.216	Vanillin	-	-	-	-	-
28.545	2,4-Di-tert-butylphenol	3.01	10.12	15.73	14.84	17.08
29.075	Eicosane	-	1.11	-	-	0.07
29.083	Decane, 3,8-dimethyl-	0.29	-	-	-	-
29.311	Vanillin	0.11	0.42	-	-	-
30.351	Hexadecane	0.21	0.54	-	-	-
30.362	Pyrrolidine, 1-(1-cyclohexen-1-yl)-	--	-	-	-	0.11
30.366	Benzaldehyde, 4-(methylthio)-	-	0.20	-	0.07	-
31.089	Eicosane	-	0.42	-	-	-
31.118	3-Methoxy-4-[3-oxo-3-(pyrrolidin-1-yl)propoxy]benzaldehyde	-	-	-	-	0.28
31.93	Dotriacontane	-	0.51	-	-	-
31.966	Tetracosane, 1-iodo-	0.19	-	-	-	-
32.073	Octadecane	-	0.55	-	-	-
32.104	Docosane, 1-iodo-	0.14	-	-	-	-
32.514	Heneicosane	0.78	3.09	-	-	-
32.554	Hexanoic acid, 4-tridecyl ester	-	-	-	0.83	1.04

**Table 3:** The elemental compositions of liquid products obtained from different catalyst contents.

Samples	Elemental Composition (wt%)				HHV (MJ/kg)
	C	H	O	N	
Lignin	61.3	6.4	29.1	1.7	20.5
0.5 wt% catalyst	63.2	6.9	26.7	0.9	23.3
1.5 wt% catalyst	65.5	6.8	25.2	0.7	24.9
2.5 wt% catalyst	66.9	6.8	24.7	0.6	27.1
3.5 wt% catalyst	69.2	7.0	21.8	0.7	30.2
4.5 wt% catalyst	68.6	7.2	22.4	0.6	29.0

**Table 4:** Weight-average (Mw) and number-average (Mn) molecular weights, and polydispersity (Mw/Mn) of lignin and solid residues (catalyst: 3.5 wt%; reaction time:40 min, 80 min, 120 min, 150 min; H_2_O_2_ concentration = 15 × 10^−2^ mol/L; T = 135 °C; pH = 3.0).

Samples	Mw (g/mol)	Mn (g/mol)	Mw/Mn
Lignin	66,671	36,726	1.81
Residues (40 min)	62,256	36,208	1.72
Residues (80 min)	41,063	27,202	1.51
Residues (120 min)	31,421	25,633	1.22
Residues (150 min)	33,468	14,597	2.29

## Data Availability

The data presented in this study are available in article.
